# Repeated Bout Rate Enhancement Is Elicited by Various Forms of Finger Tapping

**DOI:** 10.3389/fnins.2018.00526

**Published:** 2018-07-31

**Authors:** Anders Emanuelsen, Michael Voigt, Pascal Madeleine, Pia Kjær, Sebastian Dam, Nikolaj Koefoed, Ernst A. Hansen

**Affiliations:** Sport Sciences, Department of Health Science and Technology, Aalborg University, Aalborg, Denmark

**Keywords:** finger tapping rate, movement control, movement rate, voluntary movement behaviour, modulatory effects

## Abstract

Voluntary rhythmic movements, such as, for example, locomotion and other cyclic tasks, are fundamental during everyday life. Patients with impaired neural or motor function often take part in rehabilitation programs, which include rhythmic movements. Therefore, it is imperative to have the best possible understanding of control and behaviour of human voluntary rhythmic movements. A behavioural phenomenon termed repeated bout rate enhancement has been established as an increase of the freely chosen index finger tapping frequency during the second of two consecutive tapping bouts. The present study investigated whether the phenomenon would be elicited when the first bout consisted of imposed passive finger tapping or air tapping. These two forms of tapping were applied since they can be performed without descending drive (passive tapping) and without afferent feedback related to impact (air tapping) – as compared to tapping on a surface. Healthy individuals (*n* = 33) performed 3-min tapping bouts separated by 10 min rest. Surface electromyographic, kinetic, and kinematic data were recorded. Supportive experiments were made to measure, for example, the cortical sensory evoked potential (SEP) response during the three different forms of tapping. Results showed that tapping frequencies in the second of two consecutive bouts increased by 12.9 ± 14.8% (*p* < 0.001), 9.9 ± 6.0% (*p* = 0.001), and 16.8 ± 13.6% (*p* = 0.005) when the first bout had consisted of tapping, passive tapping, and air tapping, respectively. Rate enhancement occurred without increase in muscle activation. Besides, the rate enhancements occurred despite that tapping, as compared with passive tapping and air tapping, resulted in different cortical SEP responses. Based on the present findings, it can be suggested that sensory feedback in an initial bout increases the excitability of the spinal central pattern generators involved in finger tapping. This can eventually explain the phenomenon of repeated bout rate enhancement seen after a consecutive bout of finger tapping.

## Introduction

Voluntary rhythmic movement is a fundamental part of everyday human life. For example, healthy individuals perform rhythmic movement during locomotion and other cyclic tasks. As another example, patients with impaired neural or motor function are often taking part in rehabilitation programmes consisting of rhythmic movements to maintain or improve motor control (Jacobs and Nash, 2004; Schwartz et al., 2011). Thus, it is imperative that we have the best possible understanding of the human control and behaviour of voluntary rhythmic movement.

A general understanding concerning the organization of the nervous system and the function of voluntary rhythmic movement is that these are a result of interactions between supraspinal centres, spinal central pattern generators (CPGs), and sensory feedback (Dimitrijevic et al., 1998; Zehr and Duysens, 2004; Grillner, 2009). The CPGs are spinal neural networks capable of producing rhythmic patterned output without rhythmic sensory and supraspinal descending input (Hooper, 2000; Marder and Bucher, 2001). Studies performed on animals have shown that stereotyped rhythmic movement to a considerable extent is controlled by CPGs (Kiehn, 2006; Garcia-Campmany et al., 2010; Gosgnach, 2011). The inter-relationship between supraspinal descending drive, sensory feedback, and CPG-activity is largely unrevealed in humans (Dimitrijevic et al., 1998; Zehr, 2005; Hundza et al., 2012). Though, it is considered that control of rhythmic movement is similar in animals and humans (Dimitrijevic et al., 1998; Zehr, 2005; Hansen, 2015). To increase our understanding, studies applying reflex modulation during, for example, arm cycling and pedalling (Zehr et al., 2007; Hundza and Zehr, 2009), voluntary pedalling (Sakamoto et al., 2007; Hansen and Ohnstad, 2008; Stang et al., 2016), and finger tapping (Shima et al., 2011; Hansen et al., 2015; Mora-Jensen et al., 2017) have been performed. Such studies reflect that investigation of CPG-mediated voluntary rhythmic movement in humans is challenged by the restricted access to the spinal cord (Dietz, 2003; Zehr, 2005). However, it has been argued that analysis of motor behaviour can be used to increase our understanding of the nervous system’s organization and function (Goulding, 2009; Schlinger, 2015).

Index finger tapping is a rhythmic movement, which is performed in a number of daily activities such as during keyboard texting. Besides, finger tapping is a useful task in human movement science to elucidate aspects related to, for example, voluntary rhythmic movement in healthy individuals (Aoki et al., 2005; Hansen and Ohnstad, 2008; Wu et al., 2008) as well as in patients with, for example, Parkinson’s disease (Keitel et al., 2013; Teo et al., 2013). The freely chosen tapping frequency during voluntary index finger tapping has been suggested to be controlled by spinal CPGs in an inter-relationship with descending drive from supraspinal centres, as well as sensory feedback (Hansen and Ohnstad, 2008; Shima et al., 2011). Regardless of the details of the control, several aspects of the movement behaviour and control of voluntary finger tapping, for example, effects of repetition history, are largely unrevealed.

A previous study demonstrated the existence of a behavioural phenomenon termed *repeated bout rate enhancement* (Hansen et al., 2015). Briefly, the phenomenon consists of an increased freely chosen finger tapping frequency in the second of two consecutive tapping bouts separated by a rest period. Thus, it was reported that a cumulating increase of the freely chosen tapping frequency of approximately 8% occurs across four bouts of tapping, which were all separated by 10 min rest periods (Hansen et al., 2015). The finding was recently replicated (Mora-Jensen et al., 2017). Our previous studies (Hansen et al., 2015; Mora-Jensen et al., 2017) inspired us to formulate hypotheses regarding the mechanism causing repeated bout rate enhancement. It was, for example, suggested that the rate enhancement was caused by a net excitation of the spinal CPG (Finkel et al., 2014), the supraspinal centres (De Luca and Erim, 1994), or a combination of the two. In a recent review, focussing on findings from animal studies, it was suggested that sensory feedback has the potential to excite the CPG (**Figure 1** in Frigon, 2017). However, little is known about this aspect. For instance, it is unknown if sensory feedback caused by a bout of imposed passive tapping can cause rate enhancement in a subsequent bout of freely chosen tapping in the able-bodied human.

**FIGURE 1:**
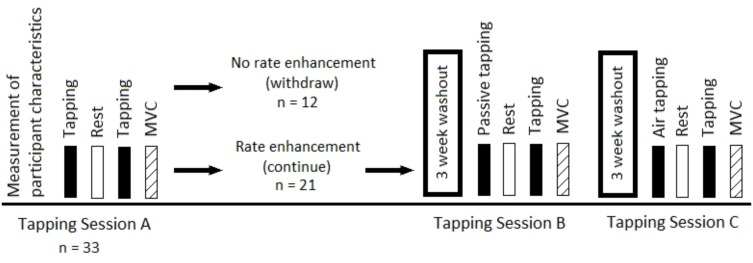
Illustration of the design of Experiment 1. The order of Sessions B and C was counterbalanced and performed in a crossover fashion.

The overall objective of the present study was to conduct a detailed investigation of the phenomenon of repeated bout rate enhancement during the task of finger tapping. More specifically, the main purpose was to test the experimental hypothesis that imposed passive tapping (i.e., imposed sinusoidal tapping-like finger movements without requirement of supraspinal drive) would also elicit repeated bout rate enhancement. An affirmative finding would support the working hypothesis that sensory feedback in itself can elicit a neural excitation and thereby cause an enhancement of the tapping frequency during voluntary stereotyped CPG-mediated finger tapping. In contrast, an unsupportive finding would support the working hypothesis that sensory feedback in itself is not causing such responses. For secondary purposes, supportive tests involving tapping-like movements in the air (termed air tapping), tapping at pre-set frequencies, and measurements of cortical sensory evoked potential (SEP) responses were also performed as part of the present study.

## Materials and Methods

### Participants

Healthy individuals volunteered to participate in the present study. For exact number of participants and their characteristics in the various parts of the study, the reader is referred to information below. The participants received written and oral information about the procedures of the study and the overall aim. The participants were not informed about the specific aims and hypotheses of the study. This was to avoid deliberate control of the performed finger tapping. Exclusion criteria were any history of neural or musculoskeletal disorders or diseases, and often execution of rhythmic tasks with their fingers, such as during playing an instrument or gaming more than an hour weekly. Participants were instructed not to consume alcohol or euphoric substances during the final 24 h before testing. In addition, they were informed not to consume coffee during the final 3 h before testing. This study was carried out in accordance with the recommendations of The North Denmark Region Committee on Health Research Ethics with written informed consent from all subjects. All subjects gave written informed consent in accordance with the Declaration of Helsinki. The protocol was approved by The North Denmark Region Committee on Health Research Ethics (N-20170017).

### Overall Design

The present study consisted of a total of three experiments, which were performed on separate occasions and in chronological order. Experiment 1 constituted the key experiment in which the main purpose was to investigate whether passive tapping would elicit the previously described phenomenon of repeated bout rate enhancement. In this experiment, air tapping was also performed. Experiments 2 and 3 could be considered supportive experiments with the purpose of supporting Experiment 1. Experiment 2 was performed to test whether the method of surface electromyography (sEMG) recording was sufficiently sensitive to detect a difference in muscle activation during tapping at two pre-set tapping frequencies, which differed by 12% (corresponding to the magnitude of rate enhancement). Experiment 3 was performed to elucidate whether various forms of tapping would result in different patterns of somatosensory feedback at the cortical level.

### Experiment 1

A total of 33 individuals (23 men, 10 women, 1.82 ± 0.04 m, 80.4 ± 12.5 kg, 25.4 ± 3.5 years, 28 right-handed, 5 left-handed) participated in Experiment 1. In total, each participant reported to the laboratory three times for Experiment 1. The first test session was considered a baseline test. With regard to this, it should be noted that the degree of steadiness of the freely chosen tapping frequency has previously been investigated. Thus, across seven tests performed over a 12-week period, an intra-individual 95% confidence interval of the tapping frequency of 13 taps min^−1^ was reported, as an average across seven individuals (Hansen and Ohnstad, 2008). The three attendances were separated by 3-week washout periods, which should ensure that the freely chosen tapping frequency had returned to baseline at each attendance (Hansen and Ohnstad, 2008). At each of the three attendances, a single test session was performed. These three test sessions are referred to as Session A, Session B, and Session C in the following. **Figure 1** illustrates the design of Experiment 1. All participants performed Session A at the first attendance for determination of the freely chosen tapping frequency. The tapping frequency found in Session A was subsequently applied in Session B. The order of Sessions B and C was counterbalanced and performed in a crossover fashion. The participant reported to the laboratory at the same time of the day for all three test sessions, to avoid potential influence of circadian rhythm on finger tapping frequency (Moussay et al., 2002). In addition, there was no warm-up before testing to prevent any form of rate enhancement before the first tapping bout.

#### Session A

Session A was initiated by determining the participant’s age, body height, and body mass. Thereafter, a demonstration of how to perform tapping and of the test procedure in general was provided. During all tests, the participant assumed a standardized test position. The participant was instructed to keep the palm of the right hand flat on the table. The participant’s back was straight while shoulder and elbow joints were flexed approximately 50° and 45°, respectively. The lower arm was resting on the table. For detailed descriptions, the reader is referred to a previous publication (Sardroodian et al., 2016). The participant performed tapping with the index finger of the right hand at a freely chosen frequency, while the remaining four fingers of the right hand were in an extended position and resting state on the table (Hansen et al., 2015; Sardroodian et al., 2016; Mora-Jensen et al., 2017). It was emphasized that the tapping should *not* be performed as fast as possible, but rather at the participant’s “own preferred rhythm” while at the same time “thinking about something else.” Besides, the participant did not receive any particular instruction about maintaining a constant tapping frequency throughout the tapping bouts. After the demonstration, the motor points of the right extensor digitorum communis (EDC) and flexor digitorum superficialis (FDS) muscles were identified by electrical stimulation with a handheld pair of electrodes from a DISA electrostimulation device (Type 9014E0102, DISA Elektronik, Herlev, Denmark). This procedure enabled to find the precise locations for placement of the sEMG electrodes considering the discrepancies concerning the electrode positioning (Zipp, 1982; Leijnse et al., 2008). With this device, single 1 ms stimuli were applied at 0.5 Hz. Stimulus amplitude varied between 1 and 80 V. The positions on the forearm eliciting clear index finger extension and flexion were marked. Before the sEMG electrodes were mounted, the participant’s skin over the identified motor points was shaved and rubbed with an alcohol swab (Alkoholswab, Mediq, Brøndby, Denmark) according to the SENIAM recommendations previously described (Hermens et al., 2000). Hereafter, two surface electrodes (Neuroline 720 Ag/AgCl, Ambu, Ballerup, Denmark) were placed over the markings, which for the EDC muscle, was approximatively at the midpoint on the dorsum of the forearm in longitudinal alignment with the direction of the muscle and separated by 2 cm. Furthermore, electrodes were placed over markings for the FDS muscle, on the volar surface of the forearm aligned longitudinally proximal to the wrist using the same inter-electrode distance. A reference electrode was placed over the lateral epicondyle of humerus. A light emitting-diode (LED) tracker, which is part of a motion capture system (Standard VZ-4000v, Phoenix Technologies Inc., Burnaby, BC, Canada), was attached to the centre of the nail of the participant’s index finger of the right hand. Eventually, the participant was comfortably seated in an office chair assuming the test position, and the test could begin.

First, background sEMG activity was recorded for 5 s while the arm rested on the table. Then, a 3-min tapping bout was performed at a freely chosen tapping frequency. During this bout, the finger tapped on a force transducer (FS6–250, AMTI, Watertown, MA, United States). Data recordings and analyses were performed across the entire tapping bout. Subsequent to the first tapping bout, the participant had a 10-min rest period. The rest period was followed by a second 3-min tapping bout, again performed at a freely chosen tapping frequency. After the tapping bouts, the participant performed three 5-s maximal voluntary contractions (MVCs) of isometric index finger flexion (i.e., pressing on the force transducer). This was followed by three 5-s MVCs of isometric index finger extension (i.e., trying to lift the finger, which was secured to the top of the force transducer by a strap). For the MVCs, the participant was instructed to gradually increase the force towards a maximum throughout the initial 3 s and thereafter maintain the force for an additional 2 s. All MVCs were separated by 1-min rest periods. At the end of Session A, the participant had a brief familiarization with the custom-built machine used to apply passive tapping in Session B (see below).

Participants who showed repeated bout rate enhancement from the first to the second bout in Session A (*n* = 21, 16 men, 5 women, 1.83 ± 0.08 m, 82.1 ± 13.2 kg, 25.3 ± 3.1 years, 18 right-handed, 3 left-handed) were selected for participation in Sessions B and C. For this selection, a criterion of a minimum increase of 3% of the freely chosen tapping frequency from the first to the second bout was applied. This criterion was determined using test–retest data of tapping frequencies from a previous study (Hansen et al., 2015) in which an average difference of 0.3 ± 3.6% was detected.

#### Session B

**FIGURE 2:**
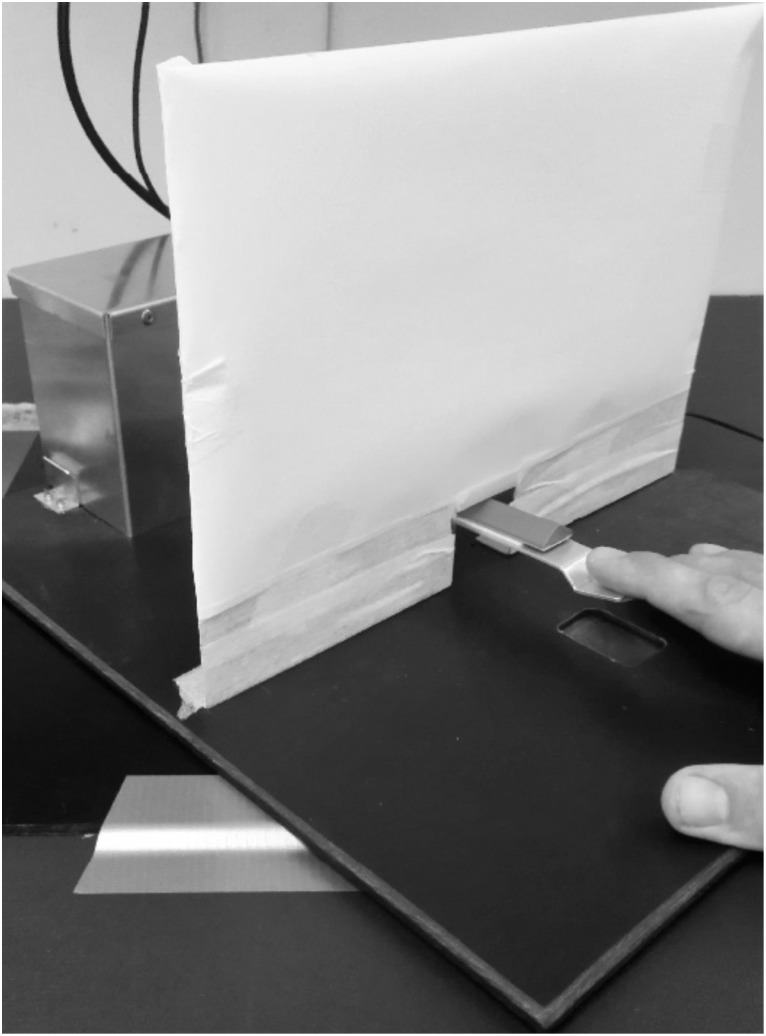
Illustration of the custom-built passive tapping machine.

Session B was initiated by demonstrating the test procedure including passive tapping during, which the participant should “relax as much as possible.” Then, sEMG electrodes and the LED-tracker were mounted as described under Session A. A 5-s background sEMG recording during rest was acquired, also as described above. Subsequently, a 3-min bout of imposed passive tapping was performed. For this part, a custom-built machine was used (**Figure 2**). Briefly, the machine consisted of a rocker arm, an electromotor (12 V DC Gearmotor, Maxon Motor, Sachseln, Switzerland) with an eccentric circular rotational wheel mounted on the axis, and a power supply that could control the movement frequency of the rocker arm. The right index finger was in a relaxed state, and the tip of the finger was placed at the end of the rocker arm. In this way, the machine could provide passive extension–flexion movement of the finger in the vertical plane. The magnitude of vertical displacement during the passive tapping was set to 24 mm, based on previous findings (Mora-Jensen et al., 2017). The exact tapping frequency during the passive tapping bout corresponded to the tapping frequency that the participant had chosen during the first tapping bout in Session A. After the passive tapping bout, a 10-min rest period followed. Subsequently, a 3-min tapping bout at a freely chosen tapping frequency was performed. At the end of Session B, the participant performed MVCs as described above.

#### Session C

In this session, tapping-like movements with the index finger was performed in the air. In the following, this is termed air tapping. The intention was to supplement the tests with a third form of tapping, which had a different pattern of somatosensory feedback compared with the tapping forms described under Sessions A and B. Thus, air tapping is characterized by the lack of input from impact on the fingertip, although proprioceptive feedback from muscle spindles is still present. Session C was initiated by demonstrating air tapping to the participant. Then, sEMG electrodes and the LED-tracker were mounted as described under Session A. A 5-s background sEMG recording during rest was acquired as the first measurement. The participant was then instructed to assume the test position in which the table supported all fingers except the index finger. The index finger had free range of motion within a hole in the table. A 3-min air tapping bout at freely chosen tapping frequency was performed. Subsequently, a 10-min rest period followed. And then, a 3-min tapping bout at freely chosen tapping frequency was performed. At the end of Session C, the participant performed MVCs as described above.

### Experiment 2

Finger tapping at pre-set frequencies was performed by 11 individuals (7 men and 4 women, 1.82 ± 0.08 m, 79.1 ± 11.6 kg, 25.5 ± 3.3 years old, 11 right-handed). These individuals consisted of a subgroup of participants from Experiment 1. In total, each participant reported to the laboratory once for Experiment 2. First, sEMG electrodes were mounted as described above. Then, the participant performed two 1-min tapping bouts at two different pre-set target tapping frequencies of 150 and 168 taps min^−1^ in a counterbalanced order. These two frequencies represented the average tapping frequencies found in the first and the second bout in Session A, Experiment 1. Thus, the average relative difference between 150 and 168 taps min^−1^ was 12%, which was similar to the observed difference in tapping frequency of 13% from the first bout to the second bout in Session A in Experiment 1. Tapping was performed on the force transducer as described above. The bouts were separated by a 2-min rest period. To obtain the target tapping frequencies, the participant followed the frequency given by a metronome.

### Experiment 3

Sensory evoked potential responses were recorded in seven participants (six men and one woman, 1.81 ± 0.07 m, 80.2 ± 8.5 kg, 28.0 ± 2.6 years old, seven right-handed). Four of the seven individuals were participants from Experiment 1. In total, each participant reported to the laboratory once for Experiment 3. The participants performed finger tapping on the force transducer, passive tapping, as well as air tapping, with tapping frequencies corresponding to 125 and 175 taps min^−1^, respectively. These two frequencies represented extremes of the observed range of tapping frequencies in Session A, Experiment 1. The participants were instructed to focus on a spot on the wall in front of them and follow the frequency provided by a blinking light from a soundless metronome. The movement of the finger was kept out of the participant’s sight during the recordings to avoid visual effects. In addition, hearing protection masked audible sound interference. Furthermore, room lighting was turned off to minimize electrical interference.

### Data Recordings and Analysis

**FIGURE 3:**
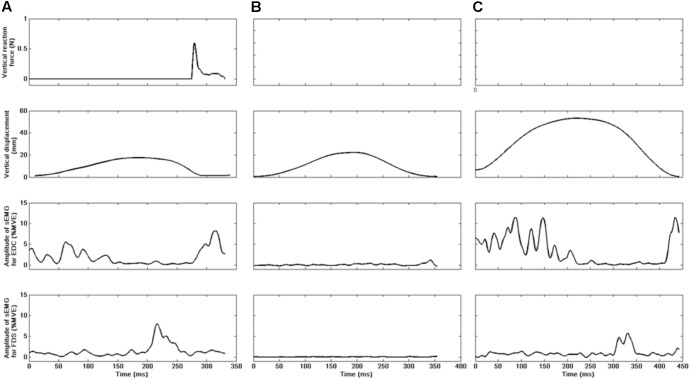
Representative recordings of individual taps in Experiment 1 from a single participant. Signals: tapping force (N), index displacement in the vertical direction (mm), as well as amplitudes of muscle activation (%MVE) for the extensor digitorum communis (EDC) and flexor digitorum superficialis (FDS) muscles. Panels **(A–C)** represent freely chosen tapping, passive tapping, and air tapping, respectively. Vertical displacement during air tapping **(C)** has been offset-adjusted. Each cycle was initiated when the tip of the finger was at its lowest point during each tap.

**Figure 3** shows a representative example of recordings of force, vertical displacement, and sEMG during the three forms of tapping performed in the present study.

#### Kinematics

The motion capture system was calibrated and set to define a 3D scaled local coordinate system. Kinematic data were sampled at 100 Hz, using VZSoft software^TM^ (Phoenix Technologies Inc., Burnaby, BC, Canada). Only the vertical movement of the fingertip in the sagittal plane provided by the LED-tracker was analysed. The analysis was performed using MATLAB version R2013a (The MathWorks, Inc., Natick, MA, United States) and a custom-written script. The maxima and the minima during the entire tapping bout were detected and vertical displacement (in mm) was calculated by subtracting the average minimum value from the average maximum value for each tap. An output trigger from the motion capture system was used to synchronize the recordings of force and sEMG.

#### Kinetics

The force transducer was checked for accuracy and linearity before the beginning of each test session, using a range of fixed loads. Tapping force was measured in the vertical direction. The force signal was amplified 4000 times, analogue low-pass filtered at 1050 Hz, and digitalized using a 12 bits NI BNC-2090A A/D-board (National Instruments, Austin, TX, United States). The force recordings were digitally low-pass filtered at 200 Hz with a fourth-order Butterworth filter and sampled at 2000 Hz using a Lab-VIEW-based (National Instruments Co., Austin, TX, United States) custom-programmed software (Mr. Kick III software, Knud Larsen, Aalborg University, Aalborg, Denmark). Force recordings were analysed using a custom-written MATLAB script. The outcome variables were calculated as averages across an entire tapping bout. The following variables were calculated for each tapping bout: (a) Tapping frequency (in taps min^−1^) was calculated as average instantaneous tapping frequency. A tapping cycle was defined as the time between two consecutive force onsets. (b) Peak force (in N) was determined as the average peak impact forces during the initial contact phase of each tap. (c) Time to peak force (in ms) was determined as the average time from the force onset to the peak force during each tap. (d) Average duration of finger contact phase (in ms) was determined as the time from the force onset to the force offset. Force onset was determined as the time point of initial finger contact. Force offset was determined as the time point where the force returned to the baseline force value. Besides, the force signals recorded during the MVC trials were further smoothed with a running average using 100 ms intervals with no overlap, and subsequently the highest force value from each trial was determined. The largest of these three force values was used for normalisation of force recorded during tapping.

#### Muscle Activation

The sEMG signals were pre-amplified 100 times with a total gain of ×1000. Then, the signals were recorded using custom-programmed software (Mr. Kick III software, Aalborg University, Aalborg, Denmark). The sEMG signals were analogue high- and low-pass filtered at 10 and 1000 Hz, respectively, and A/D converted using a 12 bits NI BNC-2090A A/D-board (National Instruments, Austin, TX, United States). Then, it was sampled at 5000 Hz. Skin-electrode impedance was kept below 5 kΩ. The sEMGs were analysed using a custom-written MATLAB script. In this script, the sEMG signals were digitally band-pass filtered [10–450 Hz] with a fourth-order Butterworth filter. For recordings of background muscle activation, root mean square (RMS) values were computed over 100 ms intervals, with no overlap, and averaged across the 5 s recording. For each MVC trial, the maximal RMS-value was also found over 100 ms intervals, with no overlap. The highest of the three maximal RMS-values for flexion and extension, respectively, were used for further calculations and termed maximum voluntary electromyography (MVE). For each single tap during tapping, RMS-values were computed over 100 ms intervals, with no overlap, and then, average values were calculated across each tap. Then, the background muscle activation was subtracted. And finally, the values were normalised with respect to the MVE-values, averaged across all taps in each 3-min bout and presented as %MVE.

#### Sensory Evoked Potential Responses

The SEP responses were recorded using custom-programmed software (Mr. Kick III software, Aalborg University, Aalborg, Denmark). The EEG epochs were recorded from a monopolar disc electrode (Standard Ag/AgCl Cup, EB Neuro SpA, Firenze, Italy) at the CP3 position in the high resolution 10–20 system (Trans Cranial Technologies, 2012) to target the area of the somatosensory cortex, which represent the fingers. The recording electrode was referenced to an electrode placed at the right earlobe and a ground reference electrode placed at the forehead. The electrode locations were rubbed with abrasive gel (Nuprep, Weaver and Company, Aurora, CO, United States), and the electrodes were secured with Ten20 conductive paste (Weaver and Company, Aurora, CO, United States). The SEP signals were amplified by a gain of ×1000 and high- and low-pass filtered at 10 and 500 Hz with a fourth-order Butterworth filter, respectively. Signals were A/D converted using a 12 bits NI BNC-2090A A/D-board (National Instruments, Austin, TX, United States) at a sampling frequency of 2500 Hz. Skin-electrode impedance was kept below 5 kΩ. The SEP averaging during the finger tapping was triggered by the rising edge on the force signal (active tapping) and on the basis of the kinematic signal (during passive and air tapping). Each cycle was initiated when the tip of the finger was at its lowest point during each tap. The analysis time window adopted was the first 100 ms after the trigger. In a custom-written MATLAB script, the EEG epochs were identified. Electrooculograms (EOGs) were rejected by visual inspection of the individual epochs and on average 297 ± 66 epochs were averaged in each situation. For visualization, the epochs from each individual tapping bout were filtered with a boxcar moving average (width: 150 epochs) and plotted using the erpimage()-command in the MATLAB toolbox EEGlab^1^. Finally, “grand averages” across participants for each situation were calculated.

### Statistical Analysis

The Shapiro–Wilk test was applied to evaluate whether data resembled a normal distribution. Student’s paired two-tailed *t*-test was used to evaluate the difference between two tapping bouts in cases of normally distributed data. In cases of not normally distributed data, Wilcoxon signed rank test was applied. Pearson’s correlation coefficient was calculated to evaluate correlation between muscle activation during passive tapping and increase in tapping frequency in the subsequent tapping bout (Session B, Experiment 1). The statistical analyses were performed using IBM SPSS 24.0 (SPSS Inc., Chicago, IL, United States). Data are presented as average ± SD, unless otherwise indicated. *p* < 0.05 was considered statistically significant.

## Results

### Experiment 1

A Shapiro–Wilk test performed on bout 1 to bout 2 differences, showed that for Session A, peak force (*p* = 0.036), time to peak force (*p* < 0.001), and duration of finger contact phase (*p* = 0.049) were not normally distributed.

#### Tapping Frequency

For all participants (*n* = 33), the tapping frequencies amounted to 139.3 ± 50.6 taps min^−1^ in the first bout and 150.0 ± 61.2 taps min^−1^ in the second bout (*p* = 0.002) in Session A. This result showed that repeated bout rate enhancement occurred on the level of the gross group of participants – before selecting participants for Sessions B and C.

**FIGURE 4:**
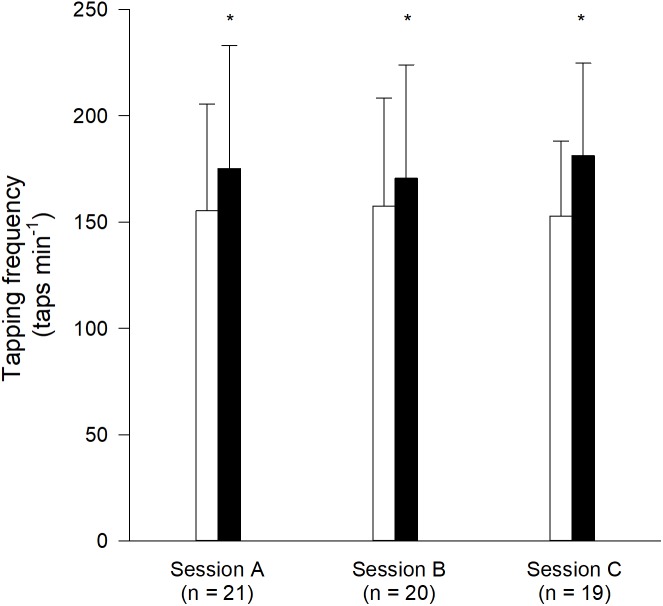
Tapping frequencies (average + SD) from Experiment 1. White bars represent the first bout in each session. Black bars represent the second bout. ^∗^Different from the first bout in Session A (*p* < 0.05). Session A included freely chosen tapping in both bouts. Session B included passive tapping in the first bout and freely chosen tapping in the second bout. Session C included air tapping in the first bout and freely chosen tapping in the second bout. In Session B, data from one participants was excluded due to muscle activation during the passive tapping bout. In Session C, data from two participants is missing due to errors during data recordings.

The tapping frequencies from the selected participants in Experiment 1 are depicted in **Figure 4**. The tapping frequency in the first bout in Session A was considered a baseline tapping frequency. The tapping frequencies in the second bouts in Session A (involving tapping), B (involving passive tapping), and C (involving air tapping) were increased by 12.9 ± 14.8% (*p* < 0.001), 9.9 ± 6.0% (*p* = 0.001), and 16.8 ± 13.6% (*p* = 0.005), respectively, as compared to the first bout in Session A. The differences in tapping frequency following passive tapping and air tapping were not statistically significantly different from the difference in tapping frequency in Session A (*p* = 0.438 and *p* = 0.348, respectively). The tapping frequency in the first bout in Session C was not different from the tapping frequency in the first bout in Session A and Session B (*p* = 0.465 and *p* = 0.377, respectively). The absolute variability of the individual freely chosen tapping frequency, reflected by the average within-bout SD, in the first and second tapping bout in Session A, was 11.4 taps min^−1^ (range of 4.0–20.2 taps min^−1^) and 12.9 taps min^−1^ (range of 4.1–23.3 taps min^−1^), respectively. For this calculation, the SD of the tapping frequency for each individual was calculated across all taps performed during the entire 3-min tapping bout. Then, the average value of all the individual SD-values was calculated across all participants.

#### Vertical Displacement

The vertical displacement of the fingertip is presented in **Table 1**, along with peak force, time to peak force, and duration of finger contact phase. The decreases in vertical displacement from the first bout in Session A to the second bout in Sessions A and B were 12.6 ± 10.5% (*p* = 0.019) and 18.6 ± 29.5% (*p* = 0.010), respectively. The difference from the first bout in Session A to the second bout in Session C of 13.7 ± 15.1% was not significant (*p* = 0.100). These differences in vertical displacement following passive tapping and air tapping were not significant from the difference in Session A (*p* = 0.780 and *p* = 0.413, respectively). The vertical displacement in the first bout in Session C was 48.5 ± 17.1 mm. This was significantly different from the first bout in Sessions A and B, with values of 24.4 ± 10.2 and 21.0 ± 0.8 mm, respectively (*p* = 0.001 and *p* < 0.001, respectively).

**Table 1:** Data from all the bouts of freely chosen tapping in Experiment 1.

	Bout 1 in Session A (baseline)	Bout 2 in Session A	Bout 2 in Session B	Bout 2 in Session C
Vertical displacement (mm)	24.4 ± 10.2	21.3 ± 9.1^∗^	19.1 ± 6.7^∗^	21.0 ± 8.6
Peak force (N)	1.02 ± 0.63	0.91 ± 0.62^∗^	0.81 ± 0.47^∗^	0.88 ± 0.57
Time to peak force (ms)	5.3 ± 2.4	5.4 ± 1.7	6.2 ± 2.4	7.0 ± 4.5
Duration of finger contact phase (ms)	107.5 ± 55.1	102.1 ± 55.5	108.2 ± 62.3	97.8 ± 49.6

*Values are presented as average ± SD. Session A included freely chosen tapping. Session B included passive tapping in the first bout. Session C included air tapping in the first bout. ^∗^Different from the first bout in Session A (baseline), p < 0.05.*

#### Peak Force

The peak force decreases from the first bout in Session A to the second bout in Sessions A and B amounted to 11.1 ± 1.1% (*p* = 0.006) and 20.2 ± 24.7% (*p* = 0.008), respectively. The difference from the first bout in Session A to the second bout in Session C of 13.5 ± 9.3% was not significant (*p* = 0.695). These differences in peak force following passive tapping and air tapping were not significant from the difference in Session A (*p* = 0.794 and *p* = 0.157, respectively).

#### Force During MVC

The force during the MVC for the EDC muscle in Sessions A, B, and C was 9.89 ± 2.73, 10.40 ± 3.16, and 10.12 ± 2.79 N, respectively. The values from Sessions B and C were not significantly different from the value in Session A (*p* = 0.082 and *p* = 0.141, respectively). The force during MVC for the FDS muscle in Sessions A, B, and C was 45.19 ± 10.03, 45.65 ± 9.19 , and 44.97 ± 10.99 N, respectively. The values in Sessions B and C were not significantly different from the value in Session A (*p* = 0.638 and *p* = 0.819, respectively).

#### Time to Peak Force

There were no significant differences between time to peak force in the first bout in Session A and the second bout in Sessions A, B, and C, respectively (*p* > 0.05). Absolute values are presented in **Table 1**.

#### Duration of Finger Contact Phase

There were no significant differences between duration of finger contact phase in the first bout in Session A and the second bout in Sessions A, B, and C, respectively (*p* > 0.05). Absolute values are presented in **Table 1**.

#### Muscle Activation

**FIGURE 5:**
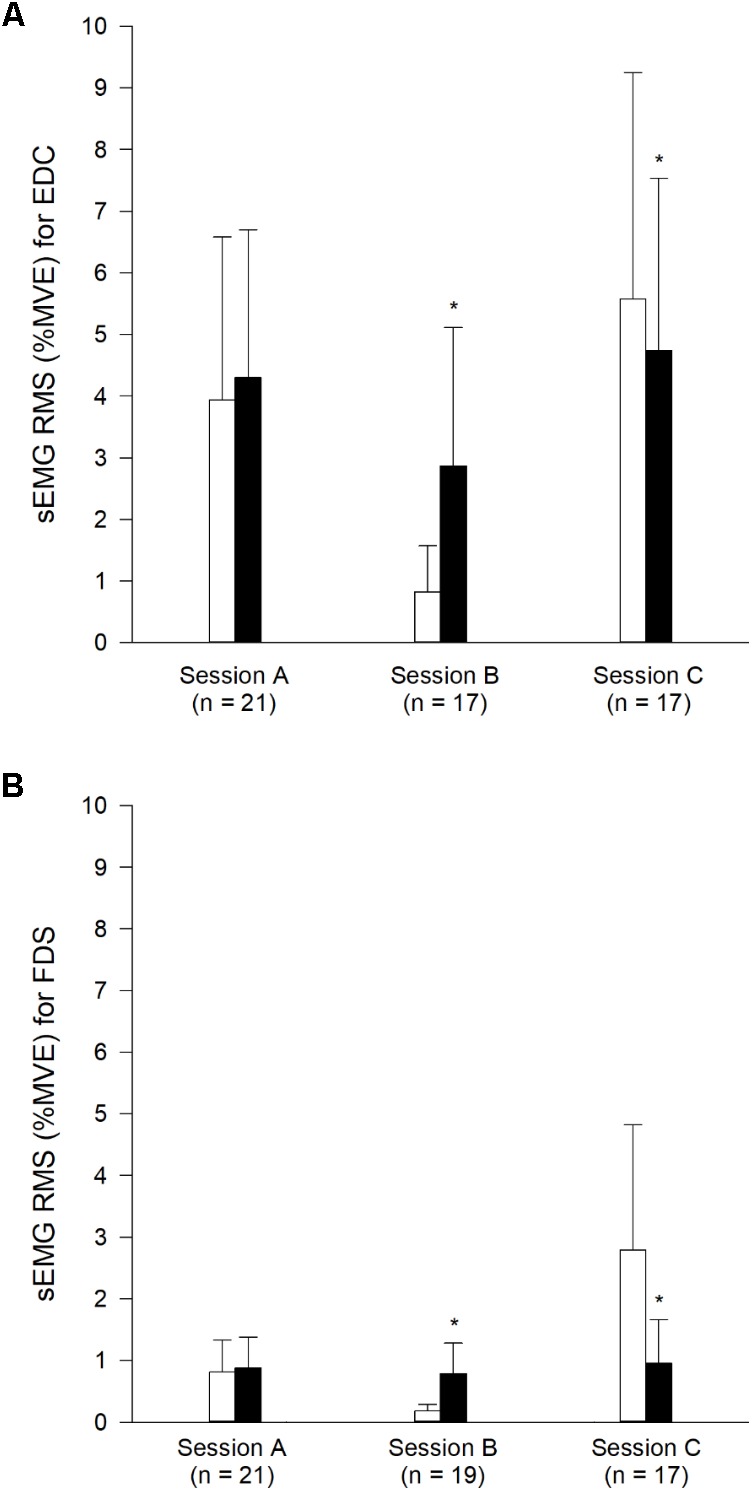
Data of muscle activation from Experiment 1 presented as average + SD %MVE. Panel **(A)** represents data for the EDC muscle. Panel **(B)** represents data for the FDS muscle. White bars represent the first bout. Black bars represent the second bout. For tapping forms in the different bouts and sessions, the reader is referred to the main text or legend of **Figure 4**. ^∗^Different from the first bout in the same session (*p* < 0.05). The varying amount of participants indicated in the tapping sessions, is a result of recording errors during EMG recordings.

**FIGURE 6:**
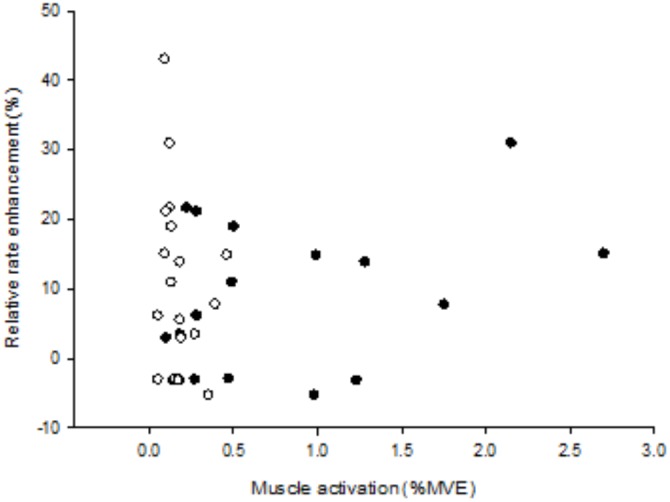
Scatter plot displaying relative rate enhancement as a function of the relative muscle activation. Relative rate enhancement was calculated as the tapping frequency in the second bout in Session B minus the tapping frequency in first bout in Session A. The difference is expressed as a percentage of the tapping frequency in the first bout in Session A. The relative muscle activation represents values from the passive tapping bout (i.e., the first bout in Session B). Filled circles represent the EDC muscle. Open circles represent the FDS muscle. The correlations were not statistically significant. For further information the reader is referred the Section “Results.” For further explanation of %MVE, the reader is referred to the Section “Muscle Activation” under the Section “Materials and Methods.”

The %MVE-values from Experiment 1 are depicted in **Figure 5**. In Session B, data from one participant was excluded due to muscle activation during the passive tapping bout. In Session C, data from two participants is missing due to errors during data recordings. For the EDC muscle, the apparent difference in %MVE from the first bout to the second bout in Session A of 9.2 ± 9.0% was not significant (*p* = 0.361). The decrease from the first bout in Session A to the second bout in Session B of 27.1 ± 15.2% was significant (*p* = 0.041). And the increase from the first bout in Session A to the second bout in Session C of 20.3 ± 5.6% was also significant (*p* = 0.019). For the FDS muscle, the differences in %MVE from the first bout in Session A to the second bout in Session A, B, and C of 7.9 ± 4.6, 2.3 ± 6.8, and 17.7 ± 36.0%, respectively, were not significant (*p* = 0.379, *p* = 0.713, and *p* = 0.138, respectively). The %MVE of the EDC and FDS muscles in the second bout in Session B were different from the first bout in Session B (passive tapping) (*p* = 0.003 and *p* < 0.001, respectively). The %MVE of the EDC and FDS muscles in the second bout in Session C were different from the first bout in Session C (air tapping) (*p* = 0.038 and *p* < 0.001, respectively). The %MVE for the EDC muscle in the first bout in Session C was significantly different from the first bout in Sessions A and B (*p* = 0.007 and *p* = 0.001, respectively). The %MVE for the FDS muscle in the first bout in Session C was significantly different from the first bout in Sessions A and B (*p* = 0.001 and *p* < 0.001, respectively). An analysis was performed to evaluate whether the relative amount of muscle activation during passive tapping (in the first bout in Session B) was correlated with subsequent relative rate enhancement (in the second bout in Session B). For this, relative rate enhancement was calculated as the tapping frequency in the second bout in Session B minus the tapping frequency in first bout in Session A. The difference is expressed as a percentage of the tapping frequency in the first bout in Session A. A scatter plot is depicted in **Figure 6**. The correlation was not significant for the EDC muscle (*p* = 0.169) as well as for the FDS muscle (*p* = 0.322).

### Experiment 2

The measured tapping frequencies were 150.6 ± 0.3 and 168.5 ± 0.2 taps min^−1^. The %MVE-values from Experiment 2 are presented in **Table 2**. For the EDC muscle, the %MVE was 9.1 ± 2.7% higher at the high tapping frequency as compared to the low tapping frequency (*p* = 0.003). For the FDS muscle, there was no significant difference between the two tapping frequencies (*p* = 0.419).

**Table 2:** Average ± SD values of muscle activation (%MVE) from Experiment 2.

	Muscle activation for the EDC muscle (%MVE)	Muscle activation for the FDS muscle (%MVE)
150 taps min^−1^	4.6 ± 2.8	1.6 ± 0.9
168 taps min^−1^	5.0 ± 2.7^∗^	1.7 ± 0.9

*EDC, extensor digitorum communis muscle; FDS, flexor digitorum superficialis muscle. Tapping was performed at pre-set target frequencies of 150 and 168 taps min^−1^. ^∗^Different from 150 taps min^−1^ (p = 0.003).*

### Experiment 3

**FIGURE 7:**
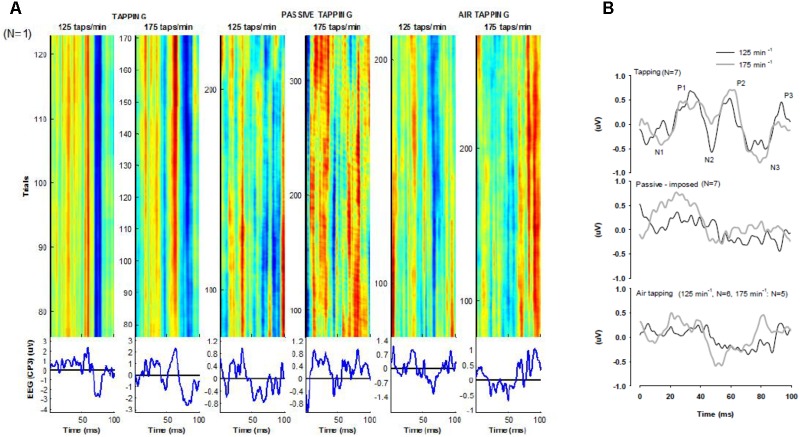
**(A)** Representative raster plots of EEG epochs (CP3-electrode) from one participant during tapping, passive tapping, and air tapping, at 125 and 175 taps min^−1^, respectively, together with averages of the involved epochs. The epochs have been subjected to a boxcar moving average filter (width 150 epochs) to reduce the noise between epochs. Dark red colour indicates higher positive amplitude, green colour indicates values close to 0, and dark blue colour indicates higher negative amplitude. **(B)** Grand averages of EEG responses during tasks of tapping, passive tapping, and air tapping, in Experiment 3. The profiles represent grand averages across 297 ± 68 artefact-free taps per tapping bout, across participants. The number of participants for each task is indicated in the figure. The time 0 ms corresponds to time of impact (during tapping) and maximal finger flexion (during passive tapping and air tapping). P1, P2, and P3 indicate successive positive peaks. N1, N2, and N3 indicate successive negative peaks.

The EEG epochs obtained during tapping at 125 taps min^−1^ demonstrated a rather systematic pattern, which is reflected both in the individual example (**Figure 7A**) and in the grand average (**Figure 7B**). The systematic pattern is observed as the continuous vertical lines during tapping at 125 taps min^−1^. The grand average in this situation demonstrated a triphasic pattern resembling the pattern reported by Hashimoto et al. (1990) elicited by air puffs to the tip of the index finger. During tapping at 175 taps min^−1^ the pattern became more variable, both intra-individually and across participants, but there is still resemblance with the pattern during tapping at 125 taps min^−1^. During passive tapping and air tapping, the responses both intra- and inter-individually became even more variable and we did not see any consistent patterns. This is observed as more incoherent vertical lines in **Figure 7A**. Additionally, the amplitude of the EEG averages during passive tapping and air tapping is 2.5–3 times smaller than the EEG averages during tapping, which indicates more asynchronous EEG signals in these situations. We assume that this overall pattern is related to differences and variability in the afferent “pictures” around the times of the trigger points and background EEG activity. During tapping, the impact of the fingertip on the rigid force transducer likely elicits a synchronous afferent burst on top of the background afferent activity generated by the movement itself, and this burst may elicit the ERPs resembling the triphasic SEPs obtained with air puffs to the index fingertip (Hashimoto et al., 1990). During passive tapping and air tapping, it is likely that no such afferent synchronous burst occurs, and that a more diffuse afferent picture is generated, which most likely is sensitive to movement variation and fluctuations in the on-going background EEG activity.

## Discussion

The present study revealed that three different types of finger tapping elicited repeated bout rate enhancement. A novel finding was that rate enhancement was elicited by passive tapping, which indicates that sensory feedback in itself can elicit repeated bout rate enhancement.

Session A showed that the freely chosen tapping frequency was higher in the second bout than in the first bout (approximately 13%). This confirmed the repeated bout rate enhancement phenomenon reported earlier (Hansen et al., 2015; Mora-Jensen et al., 2017). The relative magnitude of the rate enhancement reported in the present study was larger than previous reports of 6.0% (Mora-Jensen et al., 2017) and 8.2% (Hansen et al., 2015). This can be explained by differences in study designs. Thus, in the present study, participants who did not show rate enhancement were withdrawn. In fact, approximately 36% of the participants in the present study did not show rate enhancement. This is in line with a previous study (Mora-Jensen et al., 2017) where 33% of the participants did not show rate enhancement. Some reasons for lack of rate enhancement in an individual could include that the individual is not physiologically predisposed for the phenomenon, or that the individual arrived at the test in a rate enhanced state, or simply that random error played a role. It should be noted that the average within-bout SD-values of the tapping frequency in the first and second tapping bout in Session A (11.4 and 12.9 taps min^−1^, respectively) were smaller than the observed average rate enhancement of 20.0 taps min^−1^ (**Figure 4**). The observation, that the natural variation was smaller than the reported rate enhancement, further emphasize that the repeated rate enhancement phenomenon is larger than the within-bout variation in tapping frequency.

Session A also showed a decrease in vertical displacement of the index finger tip of approximately 13% during the rate enhanced condition. The magnitude of this decrease was comparable with a previously reported reduction (Mora-Jensen et al., 2017). Furthermore, tapping force was reduced during rate enhancement in Session A. In the study by Mora-Jensen et al. (2017), the tapping force was unaffected by rate enhancement. The reason for the discrepancy could be the difference in designs. Thus, Mora-Jensen et al. (2017) did not select individuals showing repeated bout rate enhancement for their further analysis like it was done in the present study.

The muscle activation during freely chosen tapping in Session A was on average approximately 4 and 1 %MVE for the EDC muscle and FDS muscle, respectively. With regard to the EDC muscle, the value was in line with a previously reported value of 3.5 %MVE (Lee et al., 2009). However, for the FDS muscle, Lee et al. (2009) reported a value of 8 %MVE. The larger value reported for the FDS muscle by Lee et al. (2009) can most likely be explained by differences in tapping tasks and normalization procedure. Lee et al. (2009) applied key-switch finger tapping. Furthermore, the FDS muscle is located relatively deep in the forearm with other muscles lying more superficially and resulting in a weak sEMG signal from the FDS muscle (Zipp, 1982).

The magnitude of muscle activation was not significantly different between the first and second bout in Session A. This was intuitively surprising since the tapping frequency was higher in the second bout. It has previously been reported that sEMG activity of relevant involved muscles increases during increased pre-set index finger tapping frequencies (Schnoz et al., 2000). And we confirmed that finding, for the EDC muscle, in our Experiment 2. It has been suggested that the magnitude of sEMG activity, to an extent, reflects supraspinal descending drive (Löscher et al., 1996; Carpentier et al., 2001; Arabadzhiev et al., 2010). In continuation of this, it could be speculated that the increase in sEMG activity in the EDC muscle during tapping with a pre-set rate in Experiment 2 reflected a larger supraspinal descending drive. And further, that the increased freely chosen tapping frequency in the rate enhanced condition in the second bout in Session A occurred without enhanced descending drive. Instead, it is possible that the rate enhancement occurred non-volitional, as a result of excitation of the spinal CPG (Frigon, 2017), with a modified output from the CPG (but without changed gross sEMG activity) as a result.

Session B revealed that repeated bout rate enhancement was also elicited by passive tapping. This finding supports the working hypothesis stating that sensory feedback in itself can elicit a neural excitation and thereby cause an enhancement of the tapping frequency during a subsequent bout of voluntary stereotyped CPG-mediated finger tapping. For an interpretation of the present result, three overall scenarios can be considered. First, it is possible that the rate enhancement was caused by an increased excitation of the neuronal networks of the spinal CPG. Second, it is possible that rate enhancement was caused by an excitation of supraspinal centres. Third, it is possible that rate enhancement was elicited in response to a combination of spinal and supraspinal mechanisms.

With regard to the first scenario, it is possible that the amount of peripheral sensory feedback can affect the spinal CPG’s net excitability and thereby increase CPG-mediated movement rhythm output. In animals, the excitability of spinal CPGs can be altered through pharmacological neuromodulation (Katz and Harris-Warrick, 1990; Chapman and Sillar, 2007) and electrical stimulation of afferents (Edgerton et al., 2008; Etlin et al., 2010; Finkel et al., 2014). Studies performed on spinal cord injured humans have also shown that spinal cord stimulation in combination with pharmacological neuromodulation results in excitation of the spinal circuitry (Angeli et al., 2014; Gad et al., 2017). Furthermore, it has been suggested that afferent feedback can have an excitatory effect on CPG-mediated movement output (Frigon, 2017). Therefore, we suggest that increased excitability of the spinal CPG, through activation of afferents, results in an altered intrinsic milieu within the spinal CPG. And further, that this eventually can result in enhanced motor output, observed as an enhancement of the tapping frequency. In a way, repeated bout rate enhancement might be considered as a type of “repetition priming” as described recently (Siniscalchi et al., 2016; Cropper et al., 2017). For example, it has been reported that episodic induction impacts the feeding motor programs in *Aplysia*. Cropper et al. (2017) suggested that episodic induction results in dynamic reconfiguration of CPG network activity, through intrinsic neuromodulators that exert effects that summate and persist. Furthermore, the authors suggested that the mechanisms in the feeding network of the *Aplysia* are similar to episodic behaviours in humans that dynamically alter as the mechanisms start and stop (Cropper et al., 2017).

With regard to the second scenario, it is possible that the passive tapping caused an excitation of supraspinal centres, and that this resulted in increased descending drive, and eventually an enhanced tapping frequency in the subsequent bout (De Luca and Erim, 1994; Prochazka and Yakovenko, 2007). It has previously been reported, that passive movement, compared with rest, showed activation of most of the cortical areas involved in motor control (Carel et al., 2000). It should also be noted, that it cannot be excluded that some supraspinal descending drive was present during the passive tapping bout and that this could have influenced the results. However, it should be noted that the muscle activation during passive tapping was not correlated to rate enhancement. Thus, those participants who had the most difficulty relaxing during passive tapping did not show larger rate enhancement in the subsequent bout of freely chosen tapping.

Session C showed that repeated bout rate enhancement was also elicited by air tapping. Thus, the relative difference in tapping frequency between the first bout in Session A and the second bout in Session C was comparable to the differences found for Sessions A and B. The goal of including air tapping was to omit the impact on the fingertip and thus first and foremost create a tapping form that caused a different somatosensory feedback as compared to tapping on the force transducer. The results from Experiment 3 confirmed that this goal was achieved. These results represent a qualitative analysis of the SEP responses during the various forms of tapping. The SEP responses measured when performing tapping against the force transducer were comparable to previously described SEP responses from air-puff stimulation and electrical stimulation (Hashimoto et al., 1990). For comparison, the SEP responses during passive tapping and air tapping presents altered patterns of somatosensory feedback. Despite the different patterns of somatosensory feedback to the motor cortex, repeated bout rate enhancement was elicited during all three tapping forms. This suggests that tapping frequency might be regulated independently of somatosensory feedback to the motor cortex.

Some strengths and limitations of the present study should be considered. We used electrical stimulation to position the sEMG electrodes. Still, evaluating the sEMG activity of forearm muscles is challenging. First, the EDC muscle and the FDS muscle are the primary muscles responsible for extension and flexion for the index finger. But they are also responsible for the same contractions for the middle, ring, and little fingers. Furthermore, additional muscles are involved in the extension and flexion of the index finger and thereby can influence the sEMG recordings (Arunachalam et al., 2005). In addition, the suggested locations for sEMG recordings of the EDC muscle and FDS muscle vary within the literature (Zipp, 1982; Leijnse et al., 2008). With regard to the small muscle activation during passive tapping in the present study, it is possible that even more familiarization to passive tapping could have helped the participants to better relax during passive tapping and, thus, to minimize the amount of sEMG activity during this form of tapping. This should be considered for future studies. It has been reported that stretch-reflex responses, via Ia afferents, contribute substantially (30–60%) to sEMG activity during rhythmic movement (Yang et al., 1991; Mazzaro et al., 2005). Therefore, a relatively strong contribution from peripheral afferents, especially Ia afferents, may cause non-volitional muscle activation. Other studies investigating passive finger movement have reported no sEMG activity (Reddy et al., 2001; Onishi et al., 2013; Piitulainen et al., 2013; Nakagawa et al., 2017; Sasaki et al., 2017). However, differences in tasks and sEMG recording procedure make a direct comparison challenging. Furthermore, it should be noted that the selection criterion of at least 3% enhancement of the tapping frequency for further participation in the study was based on one reference point measurement of the freely chosen tapping frequency. Multiple measurements could have been applied to establish a more solid reference point. However, since the freely chosen tapping frequency has previously been reported to be steady across weeks (Hansen and Ohnstad, 2008), this was considered redundant for the present study. Obviously, it cannot be absolutely dismissed that some of our selected participants who showed a small rate enhancement, at another test, would not have shown rate enhancement. Similarly, it cannot be absolutely dismissed that some of the participants who turned out to be merely short of being selected, would have shown rate enhancement at another test. However, as stated in the Section “Materials and Methods,” the 3% criteria was based on previously published test–retest data of the freely chosen finger tapping frequency (Hansen et al., 2015).

## Conclusion

In conclusion, the main experiment in the present study replicated the behavioural phenomenon of repeated bout rate enhancement in finger tapping. The phenomenon was also elicited in bouts of freely chosen tapping performed following bouts of passive tapping and air tapping, which represented novel findings. Supportive experiments showed that the repeated bout rate enhancement occurred in absence of a muscle activation increase. Furthermore, the rate enhancements occurred despite of different prior sensory feedback to the motor cortex. The present results are interpreted to support a working hypothesis suggesting that sensory feedback to the spinal CPG might excite this and explain the increased tapping frequency during repeated bout rate enhancement. Obviously, further studies involving, for example, more invasive techniques and animals rather than humans are required to add to the more detailed aspects of possibly involved neural mechanisms.

## Author Contributions

EH had the initial idea for the study. All authors contributed to the planning of the study design, interpretation of the results, and participated in revising of drafts to finalize the manuscript. AE performed most of the data collection assisted by PK, SD, and NK. AE and MV performed most of the data analyses assisted by EH and PM. AE completed the first draft of the manuscript.

## Conflict of Interest Statement

The authors declare that the research was conducted in the absence of any commercial or financial relationships that could be construed as a potential conflict of interest.

## Footnotes

1 https://sccn.ucsd.edu/eeglab/index.php, University of San Diego

## References

[B1] Angeli C. A. Edgerton V. R. Gerasimenko Y. P. Harkema S. J. (2014). Altering spinal cord excitability enables voluntary movements after chronic complete paralysis in humans. *Brain* 137 1394–1409. 10.1093/brain/awu038 24713270 PMC3999714

[B2] Aoki T. Furuya S. Kinoshita H. (2005). Finger-tapping ability in male and female pianists and nonmusician controls. *Mot. Control* 9 23–39. 10.1123/mcj.9.1.23 15784948

[B3] Arabadzhiev T. I. Dimitrov V. G. Dimitrov G. V. Dimitrova N. A. (2010). Interpretation of EMG integral or RMS and estimates of “neuromuscular efficiency” can be misleading in fatiguing contraction. *J. Electromyogr. Kinesiol.* 20 223–232. 10.1016/j.jelekin.2009.01.008 19233687

[B4] Arunachalam R. Weerasinghe V. S. Mills K. R. (2005). Motor control of rapid sequential finger tapping in humans. *J. Neurophysiol.* 94 2162–2170. 10.1152/jn.01173.2004 15928053

[B5] Carel C. Loubinoux I. Boulanouar K. Manelfe C. Rascol O. Celsis P. (2000). Neural substrate for the effects of passive training on sensorimotor cortical representation: a study with functional magnetic resonance imaging in healthy subjects. *J. Cereb. Blood Flow Metab.* 20 478–484. 10.1097/00004647-200003000-00006 10724112

[B6] Carpentier A. Duchateau J. Hainaut K. (2001). Motor units behaviour and contractile changes during fatigue in the human first dorsal interosseus. *J. Physiol.* 534 903–912. 10.1111/j.1469-7793.2001.00903.x 11483719 PMC2278734

[B7] Chapman R. J. Sillar K. T. (2007). Modulation of a spinal locomotor network by metabotropic glutamate receptors. *Eur. J. Neurosci.* 26 2257–2268. 10.1111/j.1460-9568.2007.05817.x 17894819

[B8] Cropper E. C. Jing J. Perkins M. H. Weiss K. R. (2017). Use of the aplysia feeding network to study repetition priming of an episodic behavior. *J. Neurophysiol.* 118 1861–1870. 10.1016/j.brainresrev.2009.08.002 28679841 PMC5599670

[B9] De Luca C. J. Erim Z. (1994). Common drive of motor units in regulation of muscle force. *Trends Neurosci.* 17 299–305. 10.1016/0166-2236(94)90064-7 7524216

[B10] Dietz V. (2003). Spinal cord pattern generators for locomotion. *Clin. Neurophysiol.* 114 1379–1389. 10.1016/S1388-2457(03)00120-2 12888019

[B11] Dimitrijevic M. R. Gerasimenko Y. Pinter M. M. (1998). Evidence for a spinal central pattern generator in humansa. *Ann. N. Y. Acad. Sci.* 860 360–376. 10.1111/j.1749-6632.1998.tb09062.x 9928325

[B12] Edgerton V. R. Courtine G. Gerasimenko Y. P. Lavrov I. Ichiyama R. M. Fong A. J. (2008). Training locomotor networks. *Brain Res. Rev.* 57 241–254. 10.1016/j.brainresrev.2007.09.002 18022244 PMC2288528

[B13] Etlin A. Blivis D. Ben-Zwi M. Lev-Tov A. (2010). Long and short multifunicular projections of sacral neurons are activated by sensory input to produce locomotor activity in the absence of supraspinal control. *J. Neurosci.* 30 10324–10336. 10.1523/JNEUROSCI.1208-10.2010 20685976 PMC6634674

[B14] Finkel E. Etlin A. Cherniak M. Mor Y. Lev-Tov A. Anglister L. (2014). Neuroanatomical basis for cholinergic modulation of locomotor networks by sacral relay neurons with ascending lumbar projections. *J. Comp. Neurol.* 522 3437–3455. 10.1002/cne.23613 24752570

[B15] Frigon A. (2017). The neural control of interlimb coordination during mammalian locomotion. *J. Neurophysiol.* 117 2224–2241. 10.1177/1073858410396101 28298308 PMC5454475

[B16] Gad P. Gerasimenko Y. Zdunowski S. Turner A. Sayenko D. Lu D. C. (2017). Weight bearing over-ground stepping in an exoskeleton with non-invasive spinal cord neuromodulation after motor complete paraplegia. *Front. Neurosci.* 11:333. 10.3389/fnins.2017.00333 28642680 PMC5462970

[B17] Garcia-Campmany L. Stam F. J. Goulding M. (2010). From circuits to behaviour: motor networks in vertebrates. *Curr. Opin. Neurobiol.* 20 116–125. 10.1016/j.conb.2010.01.002 20138753 PMC2847443

[B18] Gosgnach S. (2011). The role of genetically-defined interneurons in generating the mammalian locomotor rhythm. *Integr. Comp. Biol.* 51 903–912. 10.1093/icb/icr022 21576118

[B19] Goulding M. (2009). Circuits controlling vertebrate locomotion: moving in a new direction. *Nat. Rev. Neurosci.* 10 507–518. 10.1038/nrn2608 19543221 PMC2847453

[B20] Grillner S. (2009). Pattern generation. *Encycl. Neurosci.* 7 487–494. 10.1016/B978-008045046-9.01341-3

[B21] Hansen E. A. (2015). On voluntary rhythmic leg movement behaviour and control during pedalling. *Acta Physiol.* 214(Suppl. 702), 1–18. 10.1111/apha.12529 26094819

[B22] Hansen E. A. Ebbesen B. D. Dalsgaard A. Mora-Jensen M. H. Rasmussen J. (2015). Freely chosen index finger tapping frequency is increased in repeated bouts of tapping. *J. Mot. Behav.* 47 490–496. 10.1080/00222895.2015.1015675 25811421

[B23] Hansen E. A. Ohnstad A. E. (2008). Evidence for freely chosen pedalling rate during submaximal cycling to be a robust innate voluntary motor rhythm. *Exp. Brain Res.* 186 365–373. 10.1007/s00221-007-1240-5 18071679

[B24] Hashimoto I. Yoshikawa K. Sasaki M. (1990). Latencies of peripheral nerve and cerebral evoked responses to air-puff and electrical stimuli. *Muscle Nerve* 13 1099–1104. 10.1002/mus.880131203 2266984

[B25] Hermens H. J. Freriks B. Disselhorst-Klug C. Rau G. (2000). Development of recommendations for SEMG sensors and sensor placement procedures. *J. Electromyogr. Kinesiol.* 10 361–374. 10.1016/S1050-6411(00)00027-4 11018445

[B26] Hooper S. L. (2000). Central pattern generators. *Curr. Biol.* 10 176–179. 10.1016/S0960-9822(00)00367-5 10713861

[B27] Hundza S. R. de Ruiter G. C. Klimstra M. Zehr E. P. (2012). Effect of afferent feedback and central motor commands on soleus H-reflex suppression during Arm cycling. *J. Neurophysiol.* 108 3049–3058. 10.1152/jn.00485.2011 22956797

[B28] Hundza S. R. Zehr P. E. (2009). Suppression of soleus H-reflex amplitude is graded with frequency. *Exp. Brain Res.* 193 297–306. 10.1007/s00221-008-1625-0 19011847

[B29] Jacobs P. L. Nash M. S. (2004). Exercise recommendations for individuals with spinal cord injury. *Sports Med.* 34 727–751. 10.2165/00007256-200434110-00003 15456347

[B30] Katz P. S. Harris-Warrick R. M. (1990). Neuromodulation of the crab pyloric central pattern generator by serotonergic/cholinergic proprioceptive afferents. *J. Neurosci.* 10 1495–1512. 10.1523/JNEUROSCI.10-05-01495.1990 2332793 PMC6570082

[B31] Keitel A. Wojtecki L. Hirschmann J. Hartmann C. J. Ferrea S. Südmeyer M. (2013). Motor and cognitive placebo-/nocebo-responses in Parkinson’s disease patients with deep brain stimulation. *Behav. Brain Res.* 250 199–205. 10.1016/j.bbr.2013.04.051 23651878

[B32] Kiehn O. (2006). Locomotor circuits in the mammalian spinal cord. *Annu. Rev. Neurosci.* 29 279–306. 10.1146/annurev.neuro.29.051605.112910 16776587

[B33] Lee D. L. Kuo P. Jindrich D. L. Dennerlein J. T. (2009). Computer keyswitch force–displacement characteristics affect muscle activity patterns during index finger tapping. *J. Electromyogr. Kinesiol.* 19 810–820. 10.1016/j.jelekin.2008.03.011 18515146

[B34] Leijnse J. Campbell-Kyureghyan N. H. Spektor D. Quesada P. M. (2008). Assessment of individual finger muscle activity in the extensor digitorum communis by surface EMG. *J. Neurophysiol.* 100 3225–3235. 10.1152/jn.90570.2008 18650306

[B35] Löscher W. N. Cresswell A. G. Thorstensson A. (1996). Excitatory drive to the a-motoneuron pool during a fatiguing submaximal contraction in man. *J. Physiol.* 1 271–280. 10.1113/jphysiol.1996.sp021214 PMC1158777 9011619

[B36] Marder E. Bucher D. (2001). Central pattern generators and the control of rhythmic movements. *Curr. Biol.* 11 986–996. 10.1016/S0960-9822(01)00581-4 11728329

[B37] Mazzaro N. Grey M. J. Sinkjaer T. (2005). Contribution of afferent feedback to the soleus muscle activity during human locomotion. *J. Neurophysiol.* 93 167–177. 10.1152/jn.00283.2004 15356177

[B38] Mora-Jensen M. H. Madeleine P. Hansen E. A. (2017). Vertical finger displacement is reduced in index finger tapping during repeated bout rate enhancement. *Mot. Control* 21 457–467. 10.1123/mc.2016-0037 28001481

[B39] Moussay S. Dosseville F. Gauthier A. Larue J. Sesboüe B. Davenne D. (2002). Circadian rhythms during cycling exercise and finger-tapping task. *Chronobiol. Int.* 19 1137–1149. 10.1081/CBI-120015966 12511031

[B40] Nakagawa M. Sasaki R. Tsuiki S. Miyaguchi S. Kojima S. Saito K. (2017). Effects of passive finger movement on cortical excitability. *Front. Hum. Neurosci.* 11:216. 10.3389/fnhum.2017.00216 28515687 PMC5413571

[B41] Onishi H. Sugawara K. Yamashiro K. Sato D. Suzuki M. Kirimoto H. (2013). Neuromagnetic activation following active and passive finger movements. *Brain Behav.* 3 178–192. 10.1002/brb3.126 23531918 PMC3607158

[B42] Piitulainen H. Bourguignon M. De Tiège X. Hari R. Jousmäki V. (2013). Corticokinematic coherence during active and passive finger movements. *Neuroscience* 238 361–370. 10.1016/j.neuroscience.2013.02.002 23402851

[B43] Prochazka A. Yakovenko S. (2007). Predictive and reactive tuning of the locomotor CPG. *Integr. Comp. Biol.* 47 474–481. 10.1093/icb/icm065 21672856

[B44] Reddy H. Floyer A. Donaghy M. Matthews P. (2001). Altered cortical activation with finger movement after peripheral denervation: comparison of active and passive tasks. *Exp. Brain Res.* 138 484–491. 10.1007/s002210100732 11465747

[B45] Sakamoto M. Tazoe T. Nakajima T. Endoh T. Shiozawa S. (2007). Voluntary changes in leg cadence modulate arm cadence during simultaneous arm and leg cycling. *Exp. Brain Res.* 176 188–192. 10.1007/s00221-006-0742-x 17061091

[B46] Sardroodian M. Madeleine P. Mora-Jensen M. H. Hansen E. A. (2016). Characteristics of finger tapping are not affected by heavy strength training. *J. Mot. Behav.* 48 256–263. 10.1080/00222895.2015.1089832 26467635

[B47] Sasaki R. Nakagawa M. Tsuiki S. Miyaguchi S. Kojima S. Saito K. (2017). Regulation of primary motor cortex excitability by repetitive passive finger movement frequency. *Neuroscience* 357 232–240. 10.1016/j.neuroscience.2017.06.009 28627417

[B48] Schlinger H. D. J. (2015). Behavior analysis and the good life. *Philos. Psychiatry Psychol.* 22 267–270. 10.1353/ppp.2015.0052

[B49] Schnoz M. Läubli T. Krueger H. (2000). Co-activity of the trapezius and upper arm muscles with finger tapping at different rates and trunk posture. *Eur. J. Appl. Physiol.* 83 207–214. 10.1007/s004210000280 11104062

[B50] Schwartz I. Sajina A. Neeb M. Fisher I. Katz-Luerer M. Meiner Z. (2011). Locomotor training using a robotic device in patients with subacute spinal cord injury. *Spinal Cord* 49 1062–1067. 10.1038/sc.2011.59 21625239

[B51] Shima K. Tamura Y. Tsuji T. Kandori A. Sakoda S. (2011). “A CPG synergy model for evaluation of human finger tapping movements,” in *Proceedings of the 2011 Annual International Conference of the IEEE Engineering in Medicine and Biology Society*, Milan, 4443–4448. 10.1109/IEMBS.2011.6091102 22255325

[B52] Siniscalchi M. J. Cropper E. C. Jing J. Weiss K. R. (2016). Repetition priming of motor activity mediated by a central pattern generator: the importance of extrinsic vs. intrinsic program initiators. *J. Neurophysiol.* 116 1821–1830. 10.1152/jn.00365.2016 27466134 PMC5144695

[B53] Stang J. Wiig H. Hermansen M. Hansen E. A. (2016). Voluntary movement frequencies in submaximal one- and two-legged knee extension exercise and pedaling. *Front. Hum. Neurosci.* 10:36. 10.3389/fnhum.2016.00036 26973486 PMC4771947

[B54] Teo W. P. Rodrigues J. P. Mastaglia F. L. Thickbroom G. W. (2013). Comparing kinematic changes between a finger-tapping task and unconstrained finger flexionextension task in patients with Parkinsons disease. *Exp. Brain Res.* 227 323–331. 10.1007/s00221-013-3491-7 23686150

[B55] Trans Cranial Technologies (2012). *10/20 System Positioning Manual*. New York, NY: Trans Cranial Technologies.

[B56] Wu J. Z. An K. Cutlip R. G. Krajnak K. Welcome D. (2008). Analysis of musculoskeletal loading in an index finger during tapping. *J. Biomech.* 41 668–676. 10.1016/j.jbiomech.2007.09.025 17991473

[B57] Yang J. F. Stein R. B. James K. B. (1991). Contribution of peripheral afferents to the activation of the soleus muscle during walking in humans. *Exp. Brain Res.* 87 679–687. 10.1007/BF00227094 1783037

[B58] Zehr E. P. (2005). Neural control of rhythmic human movement: the common core hypothesis. *Exerc. Sport Sci. Rev.* 33 54–60. 15640722

[B59] Zehr E. P. Balter J. E. Ferris D. P. Hundza S. R. Loadman P. M. (2007). Neural regulation of rhythmic arm and leg movement is conserved across human locomotor tasks. *J. Physiol.* 582 209–227. 10.1113/jphysiol.2007.133843 17463036 PMC2075277

[B60] Zehr E. P. Duysens J. E. J. (2004). Regulation of arm and leg movement during human locomotion. *Neuroscientist* 10 347–361. 10.1177/1073858404264680 15271262

[B61] Zipp P. (1982). Recommendations for the standardization of lead positions in surface electromyography. *Eur. J. Appl. Physiol. Occup. Physiol.* 50 41–54. 10.1007/BF00952243

